# A MicroRNA that Regulates TLR-Mediated Fibrosis

**DOI:** 10.3390/ijms17091519

**Published:** 2016-09-09

**Authors:** Laura Duffy, Steven O’Reilly

**Affiliations:** Faculty of Health and Life Sciences, Immunology and Cell Biology group, Northumbria University, Ellison Building, Newcastle Upon Tyne NE1 8ST, UK; laura.duffy@northumbria.ac.uk

Hepatic damage can be caused by an array of factors which, if sustained, can lead to hepatic fibrosis. The mechanisms surrounding the development of hepatic fibrosis are complex with many key regulatory and inhibitory molecules at work. Once in disarray, hepatic cells can overproduce extra cellular matrix proteins (ECM) and/or inefficiently degrade ECM, leading to pathological fibrosis. Liver fibrosis is common and currently has no effective treatment. Hepatic stellate cells (HSC) are key contributors to the development of fibrosis; this, in part, is due to the activation and transformation of the HSC into myofibroblast-like cells which over-produce key ECM molecules such as Alpha Smooth Muscle Actin (α-SMA). Once the cell is differentiated to a myofibroblasts, this excessive scarring leads to loss of function and is thought to be irreversible. This shift in expression is due to epigenetic changes within the HSCs. Epigenetics is a fast moving and relatively new area of research, in which cellular changes are altered at the gene expression level, rather than alterations to the DNA sequence, by RNA molecules such as MicroRNA (miRNA). The change in phenotype does not alter the genotype of the cells, however these changes can have detrimental effects on an organism and have been found to contribute to the pathogenesis of certain disease states [1]. MicroRNAs are small RNA molecules of between 18–23 nucleotides that negatively regulate gene expression; these fine tuners of gene expression do so, mainly, by imperfect binding to the 3’UTR of their target mRNA and cause repression; less commonly they can bind the coding region in genes, thus whilst they are small molecules they can have big effects.

Here, Chen et al. looked at the role of miR-146a-5p in HSC activation mediated by Lipopolysaccharide (LPS)/Toll-Like Receptor 4 (TLR4) [2]. This miRNA has already been shown to play a role in hepatic fibrosis via the regulation of proliferation and activation of HSC [3]. LPS is a major component of the outer membrane of gram negative bacteria and has previously been shown to mediate liver fibrosis and, in vivo, likely generates from the bacterial colonising the gut, facilitated by a ‘leaky gut’ potentiated by alcohol.

However, the functional significance of miR146a-5p in hepatic fibrosis mediated by LPS/TLR4 was uncertain. The study found that treatment with LPS and the MiR-146a-5p mimic decreased the production of key inflammatory cytokines interleukin-1β, interleukin 6 and tumour necrosis factor α, whilst the opposite is true for LX2 cells (human HSC cell line) treated with the miRNA inhibitor. Further investigations indicated that miR146a-5p negatively regulated pro-inflammatory cytokines through modulation of the TLR4 pathway and that LPS-induced activation of HSC relied on TNF receptor associated factor-6 (TRAF6) and not interleukin-1 receptor associated kinase- 1 (IRAK1) and activation of the central transcription factor NF-Kβ. Treatment with the miRNA mimic also showed a decrease in the mRNA and protein expression of the myofibroblast marker α-SMA. It has been known for some time that NF-KB can mediate liver fibrosis, but global blockade of NF-Kβ is likely to have detrimental effects.

The signalling pathway which follows TLR-4 LPS induced-dimerization is critical in mounting an immune response to invading bacterial organisms. Inhibition of this signalling pathway may leave the host open to bacterial infection, which bears its own potential risk. Of course this could be overcome with the use of preventative antimicrobial treatment; however, a recent study has shown that in rats with a high cholesterol diet, the imbalance in gut microbiota, caused by certain antibiotics, can aggravate already existing liver injuries [4].

Despite the mounting evidence that dysregulation of miRNAs plays a role in the development of many diseases, its transfer to clinics has been sparse. A major contributing factor is the delivery of miRNA alongside the poor pharmacokinetics of synthetic miRNA. Here, Chen et al. (2016) have built on the knowledge that miRNA-146a-5p plays a key role in hepatic fibrosis.

The study here suggests that the use of a mimic miR-146-5p may be of benefit in liver fibrosis mediated by inflammatory insult. This is suggested to work by altering the expression of key mediators downstream of IRAK and NF-Kβ activation and thus altering key ECM genes that are activated in fibrosis. This is interesting as targeting this pathway may be important not only in liver fibrosis but in other fibrotic diseases such as systemic sclerosis where such pathways are altered. We have found that, in systemic sclerosis, NF-KB is altered and mediates skin fibrosis [5]. In such diseases, there are also altered levels of inflammatory cytokines and chemokines. Targeting these distinct organs with a miR-146-5p mimic could prove appealing, especially in the early stages of the disease where an inflammatory response primarily dominates before the preceding reparative phase. What is less clear is, if enhancing, such microRNAs would impede the normal inflammatory response needed to cease and desist bacterial dissemination. Further testing in animal models of fibrosis using synthetic miRs will yield the answers. However, barriers such as the stability, resistance to degradation and selectivity of the mimics are still to be overcome. Targeting the miRs to the correct cell and tissue can possibly be improved by conjugating to antibodies selective for specific antigen on the target cell. Furthermore, redundancy of miRs may also be a barrier to therapeutic intervention.
